# Comparative Efficacy of Transdermal Ketoprofen and Diclofenac Sodium in the Management of Post-Endodontic Pain: A Prospective In Vivo Study

**DOI:** 10.7759/cureus.91023

**Published:** 2025-08-26

**Authors:** Subiya Haque, Panna Mangat, Akansha Pushkar, Sara Trivedy, Mampi Biswas, Barkha Gupta

**Affiliations:** 1 Department of Conservative Dentistry and Endodontics, Kalka Dental College, Meerut, IND

**Keywords:** diclofenac, endodontics, irreversible, ketoprofen, pulpitis, transdermal

## Abstract

Introduction

Post-endodontic pain, often resulting from inflammation due to irreversible pulpitis, necessitates the use of effective analgesic strategies. Transdermal nonsteroidal anti-inflammatory drugs (NSAIDs) offer localized pain relief with minimal systemic side effects. This study aimed to compare the efficacy of transdermal ketoprofen and diclofenac sodium in controlling post-endodontic pain and to determine which NSAID provides superior pain relief during the early postoperative period using the Visual Analog Scale (VAS) to assess pain intensity at multiple time points.

Materials and methods

A prospective, comparative in vivo study was conducted in the Department of Conservative Dentistry and Endodontics at Kalka Dental College, Meerut, India, from June to December 2023. Forty patients (aged 18-55 years) with symptomatic irreversible pulpitis in the premolar teeth were allocated to two groups (n = 20 each) using consecutive sampling: Group 1 received transdermal diclofenac sodium patches (100 mg, Dr. Sabharwal’s Wound Care, Delhi, India), and Group 2 received transdermal ketoprofen patches (30 mg, Speedser, Troikaa Pharmaceuticals Ltd., Ahmedabad, India). Following standardized multi-visit endodontic treatment under local anesthesia, pain was assessed using the VAS (0-10 scale) at four, eight, 12, and 24 hours post-treatment. Data normality was confirmed using the Kolmogorov-Smirnov test, and intergroup and intragroup comparisons were analyzed using Mann-Whitney U and Friedman tests, respectively (p < 0.05).

Results

No significant differences in baseline VAS scores were found between the groups (p = 0.871). Ketoprofen showed significantly lower VAS scores than diclofenac at four hours (2.15 ± 0.03 vs. 2.70 ± 0.03), eight hours (1.25 ± 0.03 vs. 1.89 ± 0.03), 12 hours (0.45 ± 0.01 vs. 0.65 ± 0.02), and 24 hours (0.15 ± 0.01 vs. 0.55 ± 0.02) (p<0.001). No ketoprofen patients required rescue medication, unlike the two patients in the diclofenac group. Both groups showed significant within-group pain reduction (p = 0.001), with ketoprofen having a higher F-value (456.33 vs. 147.92), indicating more pronounced effects.

Conclusions

Transdermal ketoprofen (30 mg) outperformed diclofenac sodium (100 mg) in the management of post-endodontic pain, offering rapid and sustained relief. These findings support ketoprofen as the preferred analgesic in endodontic practice, highlighting the need for tailored pain management strategies.

## Introduction

Endodontic treatment, commonly known as root canal therapy, is a widely performed dental procedure aimed at alleviating pain and preserving teeth affected by pulpal or periapical pathologies [1]. Despite its effectiveness, post-endodontic pain remains a common clinical challenge, often resulting from inflammation caused by mechanical, chemical, or microbial irritation during the procedure. This pain, typically mild to moderate, can persist for several days post-treatment, significantly impacting patient comfort and satisfaction [2,3]. Therefore, effective pain management is a critical aspect of endodontic care, necessitating the exploration of innovative and efficient analgesic delivery methods [4].

Nonsteroidal anti-inflammatory drugs (NSAIDs), such as diclofenac and ketoprofen, are commonly used for their potent analgesic and anti-inflammatory properties [5]. These drugs inhibit cyclooxygenase (COX) enzymes, reduce prostaglandin synthesis, and thereby mitigate pain and inflammation [5]. Traditionally, NSAIDs are administered orally; however, this route is associated with systemic side effects, including GI irritation, renal toxicity, and cardiovascular risks, particularly with prolonged use [6]. To circumvent these limitations, transdermal drug delivery systems have emerged as promising alternatives [7]. Transdermal administration allows localized drug delivery and minimizes systemic exposure while maintaining therapeutic efficacy. This route enhances patient compliance by reducing the frequency of dosing and avoiding first-pass metabolism, which can degrade orally administered drugs [8].

Diclofenac, a phenylacetic acid derivative, is widely used for its rapid onset of action and efficacy in the management of acute pain. Its transdermal formulations, such as patches and gels, have shown success in delivering sustained drug release to the targeted tissues [9]. Ketoprofen, a propionic acid derivative, is another NSAID with anti-inflammatory and analgesic properties. Its lipophilic nature makes it particularly suitable for transdermal delivery because it enhances skin permeation and localized effects [10]. Although both drugs are effective in pain management, their comparative efficacy when administered transdermally for post-endodontic pain remains underexplored. This gap in the literature underscores the need for direct comparison to determine which NSAIDs offer superior pain relief with minimal adverse effects in the context of endodontic treatment.

The transdermal route offers several advantages, including controlled drug release, improved bioavailability, and reduced systemic side effects [7]. In the context of post-endodontic pain, where inflammation is often localized to the periapical region, transdermal delivery can provide targeted relief, potentially improving patient outcomes [7,11]. However, factors such as skin permeability, drug solubility, and patient-specific variables (such as skin type or sensitivity) may influence the efficacy of transdermal NSAIDs [11]. Additionally, the comparative pharmacodynamics of diclofenac and ketoprofen in transdermal formulations require further investigation to establish their relative effectiveness in this specific application [9,10].

This study aimed to compare the efficacy of transdermal diclofenac sodium and ketoprofen in controlling post-endodontic pain. Pain intensity was measured at four, eight, 12, and 24 hours post-treatment using the Visual Analog Scale (VAS) to track changes over time. This study also sought to determine which transdermal NSAID provided superior pain control during the early post-endodontic period.

## Materials and methods

This study was designed as a prospective, comparative in vivo study at the Department of Conservative Dentistry and Endodontics, Kalka Dental College and Hospital, Meerut, Uttar Pradesh, India, from June 2023 to December 2023. The study protocol adhered to the ethical standards outlined in the 1964 Declaration of Helsinki as revised in 2008. Ethical approval was obtained from the Institutional Ethical Committee of the Kalka Dental College, Meerut (approval number KDC/LTR/2023/0109). Prior to enrollment, all patients were provided detailed information about the study’s purpose, procedures, potential risks, and benefits. Written informed consent was obtained using standardized consent forms, ensuring voluntary participation and the right to withdraw at any time without any consequences. Both the diclofenac and ketoprofen patches used in the study have already been approved in India for pain relief and were administered within their approved dose and use. No new drugs, indications, or untested treatments were used.

The required sample size was determined a priori using the G*Power software (version 3.1.9.2; Heinrich Heine University, Düsseldorf, Germany). Based on an effect size of 0.8 (derived from prior research comparing VAS scores for post-endodontic pain management with diclofenac), a minimum of 20 patients per group (total of 40) was calculated to achieve 80% statistical power with an alpha error set at 0.05 [12]. This calculation was done for intergroup comparisons with a two-tailed t-test.

Eligibility criteria included patients aged 18-55 years of both sexes, diagnosed with symptomatic irreversible pulpitis in the premolar teeth of either jaw, as confirmed by clinical and radiographic examination. Diagnosis was made based on spontaneous pain, caries extending to the pulp observed on radiographs, an increased response to the cold test (Endo-Frost, Roeko, Germany), and an exaggerated response on an electric pulp tester (Parkell, New York, USA). Only medically healthy individuals who had not taken any analgesics in the preceding 12 hours were included in the study. The teeth required intact lamina dura on intraoral periapical radiographs, no periapical radiolucency, and no anatomical obstacles (e.g., canal calcifications) or anticipated procedural difficulties. The teeth had to be suitable for multi-visit endodontic treatment, and patients needed to provide written informed consent. Patients with preoperative VAS pain scores of equal to or less than 6 (mild to moderate pulpal or periapical pain) [12] were selected. Exclusion criteria included known hypersensitivity to diclofenac or ketoprofen, history of allergic reactions (such as bronchospasm, shock, and urticaria) to NSAIDs, active gastric or duodenal ulceration within the past six months, use of other NSAIDs or corticosteroids during the study, systemic diseases (such as bronchial asthma, epilepsy, inflammatory bowel disease, severe liver or renal insufficiency, dengue fever, and emotional/psychosomatic disorders), pregnancy or lactation, visibly broken or cracked teeth, dental developmental abnormalities, or teeth with periodontal conditions. The discontinuation criteria included failure to attend follow-up appointments or procedural errors such as fractured instruments.

Forty patients were allocated into two groups (n = 20 each) using a nonrandomized, consecutive sampling method based on the order of enrollment: Group 1 received transdermal diclofenac sodium patches (Dr. Sabharwal Wound Care, 100 mg, Dr. Sabharwal’s Wound Care, Delhi, India) once a day; Group 2 received transdermal ketoprofen patches (Speedser, 30 mg, Troikaa Pharmaceuticals Ltd., Ahmedabad, India) once a day. The contralateral or adjacent tooth in each patient served as the control for baseline comparison. All procedures were performed by a single operator trained using standardized endodontic protocols. Calibration involved a pilot study of 10 cases to ensure consistency in access cavity preparation, canal instrumentation, and obturation. The VAS was explained by a trained assistant, and inter-rater reliability was assessed via a test-retest method (Cronbach’s alpha = 0.85). The procedure began with patients completing a baseline VAS form (0-10 scale, 0 = no pain, 10 = worst pain ever) [13].

Local anesthesia (Lignox 2% with adrenaline, Indoco Remedies Ltd., Mumbai, India) was administered, followed by rubber dam isolation (GDC Rubber Dam Kit, GDC Fine Crafted Dental Pvt. Ltd., Hoshiarpur, India). If present, caries were excavated using tungsten carbide burs (Mani, Inc., Utsunomiya, Japan) and diamond burs (Shofu Inc., Kyoto, Japan), followed by access cavity preparation. Working length was determined using a 10 K-file (Dentsply Sirona, Ballaigues, Switzerland) with an electronic apex locator (Root ZX, J. Morita Corp., Kyoto, Japan), which was confirmed radiographically. Shaping was performed using ProTaper Gold rotary files (Dentsply Sirona, Ballaigues, Switzerland) up to F2. Irrigation was alternated between 3% sodium hypochlorite (10 mL per canal, Prime Dental Products Pvt. Ltd., Mumbai, India) and 17% ethylenediaminetetraacetic acid (EDTA, Dentsply Sirona, Ballaigues, Switzerland), with final irrigation with normal saline (Baxter India Pvt. Ltd., Gurugram, India). Recapitulation was performed using a 10 K-file after each rotary file. The canals were dried with paper points (Diadent, Cheongju, South Korea) and obturated with gutta-percha cones (Dentsply Sirona, Ballaigues, Switzerland) and a calcium hydroxide-based sealer (Calapex, Prevest Denpro, Jammu, India) using the single-cone technique. Temporary restorations were performed using Cavit (3M ESPE, St. Paul, MN, USA) with permanent composite restorations (Te-Econom Plus, Ivoclar Vivadent, Schaan, Liechtenstein) placed using a curing light (Woodpecker, Guilin Woodpecker Medical Instrument Co., Ltd., Guilin, China) at follow-up.

**Figure 1:**
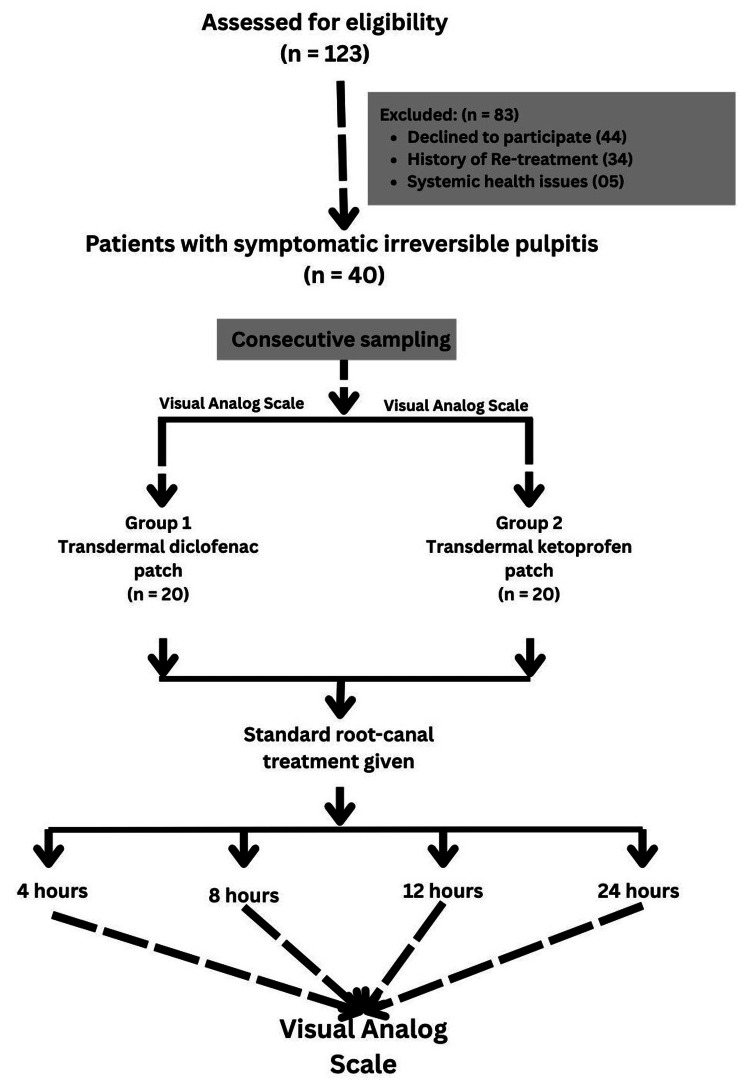
Study flowchart

Group 1 patients received a diclofenac sodium patch on the right forearm once a day on day 1. Patients in Group 2 received a ketoprofen patch in a similar manner. The patients were instructed to follow the manufacturer’s guidelines and avoid premature patch removal. Pain was assessed using the VAS, with patients marking pain intensity at four, eight, 12, and 24 hours post-treatment (Figure 1). Post-treatment pain was categorized as follows: 0 (no pain), 1 (slight pain/discomfort), 2-3 (mild pain), 4-6 (moderate to severe pain), and >6 (severe pain/swelling). Each patient was asked to report adverse effects (e.g., nausea, vomiting, dizziness, and upset stomach) and patch-related irritation. VAS forms and adverse effect reports were collected at the two-day follow-up. Patients were permitted to use rescue analgesics (e.g., paracetamol 500 mg) if their pain exceeded a VAS >6, with usage recorded and reported at follow-up.

Statistical analysis

Data were analyzed using IBM SPSS Statistics for Windows, Version 20.0 (Released 2011; IBM Corp., Armonk, NY, USA). Normality of distribution was assessed using the Kolmogorov-Smirnov test. Pain perception, measured by VAS scores, was reported as mean ± SD. For intergroup comparisons of pain perception, the Mann-Whitney U test (nonparametric test for independent samples) was used. Within-group comparisons (repeated measures) were conducted using the Friedman test (a nonparametric alternative to repeated-measures analysis of variance), followed by post hoc analysis using the Bonferroni test. Statistical significance was set at p < 0.05. The intention-to-treat (ITT) principle was used for analyses, as the ITT approach includes all patients as originally allocated, regardless of protocol deviations, to reflect real-world scenarios where patients may use rescue medications. Retaining these patients aligns with ITT, preserving the pragmatic value of the study and avoiding bias from selective exclusion.

## Results

No patch-related irritation was noted in any of the patients. No patients in the ketoprofen group required rescue medication, while two in the diclofenac group used paracetamol at eight hours. The baseline characteristics of the study groups were comparable. Age distribution was similar between males (28.25 ± 6.80 years) and females (29.15 ± 8.34 years; p = 0.671). Sex distribution did not differ significantly between the groups. These results confirmed that no demographic bias affected intergroup comparisons (Table 1).

**Table 1:** Basic characteristics of study population p > 0.05 denotes no statistical significance using an independent t-test for age and a chi-square test of independence for sex distribution in groups. Sex distribution is presented as frequency (n) and percentage (%), where n denotes the number of participants. Age is presented as mean ± SD.

Parameter	Male	Female	Test statistics	p-Value
Age (years)	28.25 ± 6.80	29.15 ± 8.34	1.02	0.671
Group 1 with diclofenac patch, n (%)	8 (40)	12 (60)	0.87	0.881
Group 2 with ketoprofen patch, n (%)	11 (55)	9 (45)		

Intergroup comparison of VAS scores revealed no significant differences at baseline (p = 0.871). However, at all postoperative intervals, Group 2 (ketoprofen) demonstrated significantly lower pain scores than Group 1 (diclofenac): four hours (2.15 vs. 2.70; p < 0.001), eight hours (1.25 vs. 1.89; p < 0.001), 12 hours (0.45 vs. 0.65; p < 0.001), and 24 hours (0.15 vs. 0.55; p < 0.001). These findings suggest that the ketoprofen patch provided superior post-treatment pain relief compared with diclofenac, with statistically significant reductions in pain intensity at every measured time point (Table 2).

**Table 2:** Intergroup comparison of VAS pain scores at multiple points with the Mann-Whitney U test ^*^p < 0.05 denotes statistical significance. Data are presented as mean and SD. VAS, Visual Analog Scale

Time interval	Group 1 with diclofenac patch		Group 2 with ketoprofen patch		Test statistics	p-Value
	Mean	SD	Mean	SD		
Pretreatment	4.71	0.34	4.56	0.38	0.41	0.871
Four hours	2.7	0.03	2.15	0.03	57.97	0.001^*^
Eight hours	1.89	0.06	1.25	0.08	28.62	0.001^*^
12 hours	0.65	0.04	0.45	0.03	17.88	0.001^*^
24 hours	0.55	0.02	0.15	0.01	80.01	0.001^*^

Table 3 shows intragroup VAS pain score comparisons using the Friedman test. In Group 1 (diclofenac patch), scores dropped from 2.70 ± 0.03 at four hours to 0.55 ± 0.02 at 24 hours (F = 147.92, p = 0.001), indicating significant pain reduction. In Group 2 (ketoprofen patch), scores decreased from 2.15 ± 0.03 at four hours to 0.15 ± 0.01 at 24 hours (F = 456.33, p = 0.001), also showing a significant reduction. Both patches effectively reduced pain, with ketoprofen showing a higher F value, suggesting more pronounced changes.

**Table 3:** Intragroup comparison of VAS pain scores using the Friedman test ^*^p < 0.05 denotes statistical significance. Data are presented as mean ± SD. VAS, Visual Analog Scale

Groups	Four hours	Eight hours	12 hours	24 hours	F value	p-Value
Group 1 with diclofenac patch	2.70 ± 0.03	1.89 ± 0.06	0.65 ± 0.04	0.55 ± 0.02	147.92	0.001^*^
Group 2 with ketoprofen patch	2.15 ± 0.03	1.25 ± 0.08	0.45 ± 0.03	0.15 ± 0.01	456.33	0.001^*^

Post hoc analysis of the diclofenac group revealed significant pain reduction at all time points (p < 0.0001), except at 12 hours vs. 24 hours (p = 0.102). The most pronounced differences occurred at early intervals (four hours vs. 12 hours / 24 hours: p < 0.0001), demonstrating rapid analgesic onset (Table 4).

**Table 4:** Pairwise comparison of pain scores using the post hoc Bonferroni test for the diclofenac group ^*^p < 0.05 denotes statistical significance.

Comparison	Mean difference of pain scores	Test statistics	p-Value
Four hours vs. eight hours	0.81	27.1	0.0001^*^
Four hours vs. 12 hours	2.05	68.3	0.0001^*^
Four hours vs. 24 hours	2.15	71.7	0.0001^*^
Eight hours vs. 12 hours	1.24	41.3	0.0001^*^
Eight hours vs. 24 hours	1.34	44.7	0.0001^*^
12 hours vs. 24 hours	0.1	3.3	0.102

The ketoprofen group showed significant pain reduction across all time intervals (p < 0.001), including at 12 hours vs. 24 hours (p = 0.001). Unlike diclofenac, ketoprofen demonstrated continuous efficacy beyond 12 hours, with progressively lower pain scores. The strongest effects occurred early (four hours vs. 24 hours, p < 0.0001), indicating a rapid onset (Table 5).

**Table 5:** Pairwise comparison of pain scores using the post hoc Bonferroni test for the ketoprofen group ^*^p < 0.05 denotes statistical significance.

Comparison	Mean difference of pain scores	Test statistics	p-Value
Four hours vs. eight hours	0.9	30	0.0001^*^
Four hours vs. 12 hours	1.7	56.7	0.0001^*^
Four hours vs. 24 hours	2	66.7	0.0001^*^
Eight hours vs. 12 hours	0.8	26.7	0.0001^*^
Eight hours vs. 24 hours	1.1	36.7	0.0001^*^
12 hours vs. 24 hours	0.3	10	0.001^*^

## Discussion

The findings of this study highlight the effectiveness of transdermal NSAIDs in managing post-endodontic pain, with ketoprofen demonstrating greater pain relief than diclofenac across multiple time points. This finding is in agreement with those of previous studies by Bhargava et al. [14] and Porwal et al. [15]. The superior performance of the ketoprofen patch can be primarily attributed to its pharmacokinetic advantages over diclofenac. Ketoprofen, with a lower molecular weight (approximately 254 Daltons) than diclofenac (296 Daltons), exhibits enhanced skin permeability, facilitating faster and more efficient transdermal absorption [16]. This molecular difference allows ketoprofen to achieve higher plasma concentrations more rapidly, leading to a quicker inhibition of prostaglandin synthesis at the site of inflammation. In contrast, the larger molecule of diclofenac may result in slower diffusion through the stratum corneum, potentially delaying its analgesic effects [17]. Studies on topical NSAID formulations have confirmed that ketoprofen preparations demonstrate superior skin permeation rates compared to other NSAIDs, including diclofenac, which correlates with improved clinical outcomes in pain management [14-16]. Furthermore, the lipophilic nature of ketoprofen enhances its partitioning into lipid-rich tissues, such as inflamed dental pulp remnants, amplifying its local anti-inflammatory action without relying solely on the systemic circulation [17]. The transdermal patch facilitates a prolonged release of ketoprofen for 24 hours, thereby improving patient adherence in contrast to topical formulations such as creams, gels, and sprays, which frequently require several daily applications [18].

Mechanistically, both drugs act as nonselective COX inhibitors, reducing the production of pro-inflammatory mediators such as prostaglandins E2 and I2, which are implicated in post-endodontic hyperalgesia [19]. However, the dual inhibition of the COX and lipoxygenase pathways provides an additional edge, mitigating leukotriene-mediated inflammation more effectively than diclofenac, which primarily targets COX enzymes [17]. This broader anti-inflammatory profile may explain the more pronounced and sustained pain reduction observed with ketoprofen, particularly in the early postoperative period when nociceptive sensitization peaks due to surgical trauma during root canal instrumentation and obturation. The transdermal route bypasses first-pass metabolism, maintains steady drug levels, and minimizes GI side effects, which are common with oral NSAIDs. This delivery method ensures a controlled release, with ketoprofen patches often achieving peak plasma levels within two to four hours, aligning well with the timing of maximal post-endodontic discomfort [20]. Modi et al. [12] investigated the effectiveness of oral versus transdermal diclofenac in alleviating pain in patients with symptomatic irreversible pulpitis after a single-visit root canal therapy. The research findings indicated that transdermal diclofenac patches demonstrate superior efficacy compared to oral diclofenac tablets in managing pain after single-visit root canal procedures after 24 hours.

For instance, research evaluating transdermal ketoprofen versus diclofenac patches in post-orthodontic extractions has shown that ketoprofen offers better analgesia, with patients requiring fewer rescue medications [10]. Similarly, in mandibular fracture surgeries, ketoprofen transdermal patches provided longer durations of pain relief and faster onset than diclofenac, attributed to its superior bioavailability [20]. A randomized trial on post-endodontic pain management echoed these results, where ketoprofen patches outperformed diclofenac in reducing VAS scores, likely due to enhanced local tissue penetration and reduced systemic variability [15]. The potency difference is further evidenced by the higher affinity of ketoprofen for COX-1 and COX-2 isoforms, enabling effective pain control at lower doses (30 mg in this study) than diclofenac (100 mg), which may require higher concentrations to achieve comparable inhibition [17].

The advantages of transdermal administration extend beyond pharmacokinetics to patient compliance and safety. By avoiding oral intake, these patches reduce the risk of gastric irritation, a common concern with NSAIDs, especially in patients with a history of ulcers, a factor that led to the strict exclusion criteria in this study [19,20]. Literature reviews on transdermal NSAIDs in dental pain emphasize their role in minimizing systemic exposure while targeting peripheral nociceptors, resulting in fewer side effects, such as nausea and dizziness [21,22]. In endodontic scenarios, where pain arises from localized inflammation in the periapical region, transdermal delivery ensures sustained drug release over 24 hours, matching the natural resolution of acute pulpal inflammation [14-16]. This is particularly beneficial in multi-visit treatments, as used here, in which repeated instrumentation could exacerbate discomfort without adequate analgesia.

Despite these strengths, the observed differences may stem from formulation-specific factors. The ketoprofen patch (Speedser, 30 mg) likely incorporates enhancers, such as menthol or oleic acid, which are common in commercial products, to boost permeation, whereas diclofenac patches (Dr. Sabharwal, 100 mg) might rely on simpler matrices. Pharmacokinetic evaluations in an animal model have shown the transdermal bioavailability of ketoprofen to be around 70-80%, higher than diclofenac’s 50-60%, supporting faster therapeutic levels [23]. Human studies corroborate this, with ketoprofen achieving steady-state concentrations more rapidly, which could explain the continuous efficacy beyond 12 hours in the ketoprofen group, unlike diclofenac, where pain stabilization occurs earlier [14-16].

Clinical implications

The superior analgesic efficacy of transdermal ketoprofen over diclofenac in managing post-endodontic pain suggests its potential as a preferred first-line option in clinical endodontics, particularly in patients with symptomatic irreversible pulpitis. This approach offers rapid onset and sustained relief, minimizing the need for rescue medications and enhancing patient comfort during the critical early post-treatment period. Transdermal delivery improves compliance through self-administration and reduces GI risks associated with oral NSAIDs, making it ideal for individuals with contraindications, such as peptic ulcers, or those preferring nonoral routes. Clinicians can integrate ketoprofen patches into multi-visit endodontic protocols to optimize pain control, potentially improving the overall treatment outcomes and patient satisfaction. Furthermore, this supports the broader adoption of topical NSAIDs in dental practice, aligning with evidence from similar trials emphasizing reduced systemic exposure while targeting peripheral inflammation effectively.

Limitations

This study has several limitations that warrant cautious interpretation of results. The nonrandomized, consecutive sampling method may introduce selection bias despite comparable baseline demographics, potentially affecting the generalizability of the findings. The small sample size and single-center design limit external validity, as the outcomes may vary across diverse populations or clinical settings. Pain assessment was restricted to 24 hours post-treatment, overlooking the long-term effects or flare-ups beyond this period. Additionally, while adverse effects were monitored, the study did not deeply explore long-term safety, necessitating further research with larger, randomized, blinded trials to confirm the efficacy and address these gaps.

## Conclusions

This study demonstrated that transdermal ketoprofen provided significantly greater pain relief than transdermal diclofenac sodium in managing post-endodontic pain associated with symptomatic irreversible pulpitis in the premolar teeth, as assessed by VAS scores at four, eight, 12, and 24 hours post-treatment. The superior efficacy of ketoprofen, particularly its rapid onset and sustained effect, highlights its potential as the preferred analgesic option in endodontic practice. These findings underscore the value of transdermal NSAIDs for effective pain control, emphasizing the need for tailored analgesic strategies and patient counseling to optimize the postoperative outcomes. Future research should incorporate randomized designs and assess rescue medication use to validate these results in diverse clinical settings.
